# Heat Transfer in Straw-Based Thermal Insulating Materials

**DOI:** 10.3390/ma14164408

**Published:** 2021-08-06

**Authors:** Dániel Csanády, Olivér Fenyvesi, Balázs Nagy

**Affiliations:** Department of Construction Materials and Technologies, Budapest University of Technology and Economics, 3 Műegyetem Rakpart, 1111 Budapest, Hungary; csanady.daniel@edu.bme.hu (D.C.); fenyvesi.oliver@emk.bme.hu (O.F.)

**Keywords:** natural fibers, raw barley and wheat straw, heat transfer, thermal conductivity, porosity, physical properties

## Abstract

An analytic-empirical model was developed to describe the heat transfer process in raw straw bulks based on laboratory experiments for calculating the thermal performance of straw-based walls and thermal insulations. During the tests, two different types of straw were investigated. The first was barley, which we used to compose our model and identify the influencing model parameters, and the second was wheat straw, which was used only for validation. Both straws were tested in their raw, natural bulks without any modification except drying. We tested the thermal conductivity of the materials in a bulk density range between 80 and 180 kg/m^3^ as well as the stem density, material density, cellulose content, and porosity. The proposed model considers the raw straw stems as natural composites that contain different solids and gas phases that are connected in parallel to each other. We identified and separated the following thermal conductivity factors: solid conduction, gas conduction in stem bulks with conduction factors for pore gas, void gas, and gaps among stems, as well as radiation. These factors are affected by the type of straw and their bulk density. Therefore, we introduced empirical flatness and reverse flatness factors to our model, describing the relationship between heat conduction in stems and voids to bulk density using the geometric parameters of undisturbed and compressed stems. After the validation, our model achieved good agreement with the measured thermal conductivities. As an additional outcome of our research, the optimal bulk densities of two different straw types were found to be similar at 120 kg/m^3^.

## 1. Introduction

The amount of built-in thermal insulations is continuously growing these days [1] due to thermal insulations usually applied to newly built and refurbished buildings because of the strict energy performance regulations that have come into force in recent years to decrease carbon emissions and energy use. In 2019, households represented 26% of the final energy consumption of the EU, of which 64% is from space heating [2]. The energy demand of buildings can be reduced by thermally insulating the houses. Nowadays, even the previously insulated buildings should receive additional thermal protection to meet regulations or repair previous building construction mistakes that cause defects and mechanical, chemical, environmental, or hygrothermal deteriorations, or accelerate natural aging [3,4,5,6].

The manufacturing energy consumption and carbon emission of materials also need to be addressed. Artificial materials usually have higher manufacturing energy consumption and higher carbon emission than natural-based thermal insulations. With the carbon sequestration considered in the calculations, the embodied carbon content of a building construction insulated by natural thermal insulations can achieve negative carbon emissions compared to artificial insulations such as mineral wool, expanded polystyrene, or polyurethane foam. This is possible because cellulose-based insulations′ embodied carbon contents are lower than those of artificial insulations [7] due to their composition or lower densities. In addition, carbon is locked in the natural insulations for the service life of the building; hence, it is sequestered [8]. The fact that using natural construction material can reduce CO_2_ emission is proven, considering the temporary carbon storage, which has a real physical impact at a 100 year time horizon [9].

All stakeholders must reduce their environmental footprint as well as their products′ energy demand, even if there are no other reasons behind it, just the financial implications of the higher energy consumption [10,11]. Today′s society also feels responsible for reducing waste by reusing or recycling materials. Therefore, the construction industry must also respond to this challenge; otherwise, they risk turning themselves into enemies of humanity in the long term [12]. One solution to reduce the amount of waste is to increase the number of natural materials in the construction industry that are biodegradable and do not leave waste behind or can be recycled after their end of life [13,14]. The use of natural-based materials and agricultural waste in construction material applications as alternative materials [15,16] or the development and use of natural-based thermal insulation materials, especially from renewable or by-product sources [17,18], are reasonable solutions to reduce our environmental footprint.

Such materials are, among several other used natural alternatives such as bark, cellulose, hemp, kenaf, flax, feathers, sheep wool, or wood fibers [7,19,20,21], the natural vegetable fiber-based thermal insulations that are gaining ground in the construction market [22] because demand for green building materials is rising sharply [23]. Among natural fibers, one of the most researched materials is the use of straw bales as building materials [24,25] because of their low thermal conductivity and high specific heat capacity, and hence low thermal diffusivity and good thermal insulation capability, especially with fibers randomly oriented and perpendicular to heat flow, and low environmental impact if the straw bale selection used for the straw-based building is suitable [26]. In recent years, regarding the thermal performance of straw-based materials, Sabapathy and Gedupudi created simplified equations to predict the thermal conductivity of straw bale constructions based on their fiber orientation, density, temperature, and relative humidity [27]. Piégay et al. used a self-consistent modeling technique to predict the equivalent thermal conductivity of vegetal-based fibrous thermal insulations as a function of thermal conductivities of solid and liquid phases and material porosity. Platt et al. [28] investigated the effect of fiber orientation on the thermal conductivity of straw bales. Yang et al. [29] investigated the thermal transmittance of straw bale walls and the effects of different structural details concerning straw bale joints; using the guarded hot box method, Conti et al. [30] realized a metering chamber within a climate chamber to be able to measure and calculate rectangular straw-bale sample′s thermal conductivity, while Costes et al. [31] used a specific guarded hot plate which was designed to measure straw bale samples of up to 50 cm thickness. Cornaro et al. [32] confirmed in their study that a straw-based natural multi-sheet wall package (named straw wall) could comply with the limited values of the thermal transmittance in Italy. Furthermore, in research examining the potential of straw as a paneled insulating material, it was found that the use of straw as a construction material can reduce the embodied energy by 50% compared to traditional masonry [33]. Despite the few existing examples, the volume of research on natural-based thermal insulation materials is far that conducted on their artificial counterparts [34]. This may be due to the properties of the artificially manufactured materials being well known and more homogenous, and having design guidelines, standards, and reliability assessments, and, despite the excellent performance of straw-based materials, there are often questions about their durability, fire risk, questionable natural anti-fungal properties and bacterial activity, and higher moisture absorption capability. The possibility of fungi/mold settling has been studied by Tobon et al. [35], and, especially for straw bales used in building envelopes, by Thomson and Walker [36]. At the same time, Yin et al. recently investigated the durability of straw bale walls in warm, humid continental climates and found that hot and humid summer climates have insignificant impacts on the durability of straw bales within straw bale walls [37]. Fire resistance of straw-based thermal insulation boards was also examined previously, and, with special binder composition, it is possible to achieve excellent performance [38]. Another issue could be that, in the case of natural materials, that most studies show higher variability of their physical properties [39,40]. However, a recent study shows that the variability of long natural-based fibers composites is in the same order of magnitude as that of carbon fiber composites [41]. Shi et al. [42] proved that natural and bio-based materials could have a good effect on health because a thermal insulation board made of straw can significantly dampen the increase in humidity level and improve the moisture environment of the inner spaces, especially in summer. These effects have also been observed in straw bale walls; however, these walls can also dampen the temperature fluctuation [43]. In the case of raw materials, we must handle structures created by nature. Still, with different natural processes, the physical properties can be modified to better meet the requirements. In many cases, there arises the problem that natural-based thermal insulators are not fire-resistant, and do not simultaneously have the lowest possible thermal conductivity and high load-bearing capability, but they have homogeneity problems or contain hazardous additives [44].

For solving these problems, our research investigates straw as a raw material in an experimental and theoretical aspect. The first step is to understand the thermal conductivity of the raw and dry fibers in their absolute natural state. The most common types of thermal insulations are inorganic fibrous materials and organic foams. In the first group, atmospheric air is hardly moving among the fibers because of friction, while in the case of organic foams, the air is embodied in the pore formation of the material. Using natural vegetable-based raw materials, fibrous thermal insulations can be created. In our study, we deal with straw-based construction materials with randomly oriented fibers, although most of the fibers are perpendicular to the heat flow direction. Papadopoulos and Anastaselos [45] dealt with heat transfer phenomena in randomly oriented fibrous insulating materials. That research focused on mineral wools, which consist of inorganic, dense, and homogeneous fibers with a relatively uniform and very small diameter. That study examined the mineral wools in a wide temperature range between 15 °C and 1000 °C. In their model, the convection was neglected because this component was too small (and constant) compared to the others. The created physical model precisely estimated the experimental results of fibrous thermal insulations. Xie et al. [46] dealt with the heat transfer in porous fibrous structures. This heat transfer model involved coupling the heat conduction with the thermal radiation in the case of porous fibrous structures. The randomly distributed fibrous media and directionally distributed fibrous media are theoretically and numerically investigated. Besides these, the study considers the connections of the heat-conducting media to each other (parallel, serial). Hoseini et al. [47] investigated heat transfer in aerogel blankets. Their paper demonstrates a theoretical and experimental study on the effective thermal conductivity of aerogel-fiber composites. The analytical model represents aerogel composites with a unit cell consisting of a cylindrical fiber surrounded by a packed bed of aerogel particles. This material structure is similar to the natural stems that are investigated in the present paper. The two cases are inverse to each other because, in straw stems, the pores are inside the stem wall, not on the surface of the fiber. These above-mentioned papers were used as starting points to develop our contribution. However, the models were created for mineral wools and aerogel blankets, but the equations prescribed for these materials had to be changed or supplemented to be able to describe the presented straw material properly. For example, a straw model needs a more accurate material property description because of the nature of the stems and because some of the natural state or loose-filled thermal natural thermal insulations are especially sensitive to bulk density changes [20,48,49,50]. In the case of the first base model [45], the material density and the bulk density of the insulation were sufficient because the glass fibers are both solids. However, the influence of the density and porosity of the heat transfer in natural-based materials also depends on the type of raw materials as well as the size and orientation of fibers, particles, or grains [19,51]. In the case of straw stems, one more density parameter must be introduced to take into consideration the porosity of the stems and the gaps among the stems. Besides stem density and material density, we must know certain physical properties, too, such as the porosity, cellulose content, and the average stem diameter, to be able to create the model for straw. During our experiments, a natural fiber length and diameter distribution were used. Therefore, in the set of stems, relatively short and long fibers occurred in the same sample, respectively. This relatively significant deviation is also true for the unit mass of fibers, and for the other parameters due to the natural origin of the material. These parameters could be handled in the model if the average of many measurements were considered.

The raw straw that is investigated in this paper is slightly different from the previously mentioned fibers because every straw stem has porosity that is similar to that of foams. Therefore, our paper deals with the modeling of heat transfer in straw-based materials, such as randomly oriented straw bale walls or straw-based fibrous thermal insulating materials. To create an accurate model, the dual nature/material complexity must be considered. Although the discussed model is a physical model, some of the material properties must be specified experimentally. Straw is a lignocellulose material; therefore, the vegetable fibers are natural composites consisting of three main base components: cellulose, lignin, and hemicellulose. Cellulose fibers provide strength, lignin is the embedding matrix, and hemicellulose is the interface that ensures the bond between the cellulose and lignin [52,53]. Wheat straw (*Triticum* spp.) is composed of 42.9% cellulose, 28.9% hemicellulose, 21.6% lignin, and only 6.6% other components, according to [54]. These ratios of the components are different by species of the grain and strongly affected by the environment (e.g., weather and soil); therefore, in our study, the ratios need to be specified experimentally. However, since the thermal conductivity of cellulose is 1.04 W/m·K transversal to fiber and 0.26 W/m·K parallel to fiber, while the thermal conductivity of hemicellulose and lignin are both isotropic and have 0.34 W/m·K thermal conductivity, we only need to separate the cellulose, which provides a sufficient approximation.

In terms of handling or describing the model, it was more expedient if the insulation was considered as a fibrous material. Because, at the macro scale, the specimens are made up of fibers (stems), the already existing fibrous material models can be used as the base of the model. The foam nature of the stem wall is taken into consideration within the model, where the thermal conductivity of solid and gas parts is combined. In our study, the above-mentioned models and literature were used as an initial basis, although they were modified in several details because of the nature of the straw fibers.

## 2. Materials and Methods

### 2.1. Raw Straw Bulk Materials

In the present research, two types of straw were investigated: barley (Type-1) and wheat (Type-2). They were grown in a different region of Hungary but collected from the same harvest period and year (Autumn 2019). Because of the different species, there were some natural disparities in the structure of stems. We used a scanning electron microscope (Phenom XL Desktop SEM, Phenom-World B.V., Eindhoven, The Netherlands) to investigate the pore microstructure of the stems.

**Figure 1:**
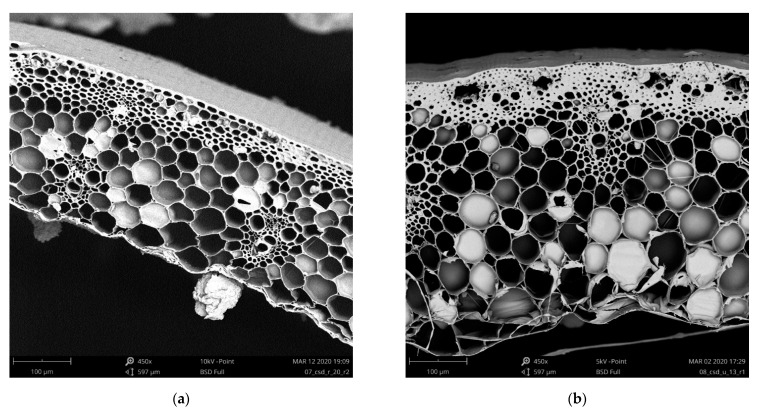
SEM images in 450× magnification of: (**a**) Type-1 straw stem; (**b**) Type-2 straw.

Based on Figure 1, it is visible that Type-1 had a thinner stem wall, smaller pores but thicker walls in the parenchyma, and thin and organized epidermis, while Type-2 had a thicker stem wall, larger pores and thinner walls in the parenchyma, and thicker epidermis; therefore, their pore composition is different. The barley and wheat arrived at the laboratory in different conditions. Type-1 was in big bales; therefore, most of the stems were compressed, but it contained intact stems too. Type-2 was only slightly compressed; therefore, almost all stems were intact.

After their arrivals, both straw types were stored under the same environmental conditions (23 ± 5 °C, 50 ± 10% relative humidity) in the laboratory until and during the tests, and the samples were conditioned for at least one month before the experiments were conducted to reach equilibrium with the laboratory environment.

### 2.2. Methods

#### 2.2.1. Physical Properties Measurement

**Figure 2:**
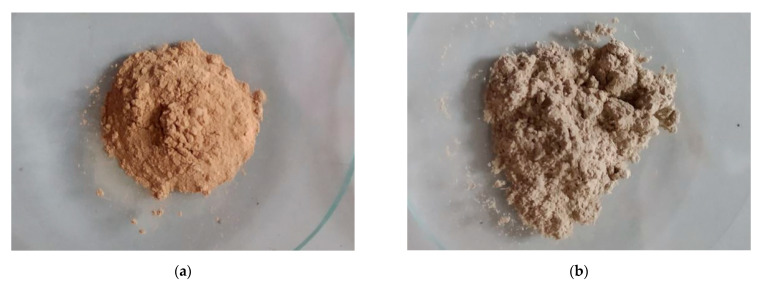
Milled straw dust of: (**a**) Type-1 straw; (**b**) Type-2 straw.

The physical properties of the tested stems were investigated to contribute to the heat transfer model. To measure material density, firstly, some randomly sampled stems from the straw bales were dried at 120 °C for 48 h. After the drying process, an Labor MIM LE-101 ball mill (Labor Műszeripari Művek, Budapest, Hungary) was used to mill the stems to dust (see Figure 2).

**Figure 3:**
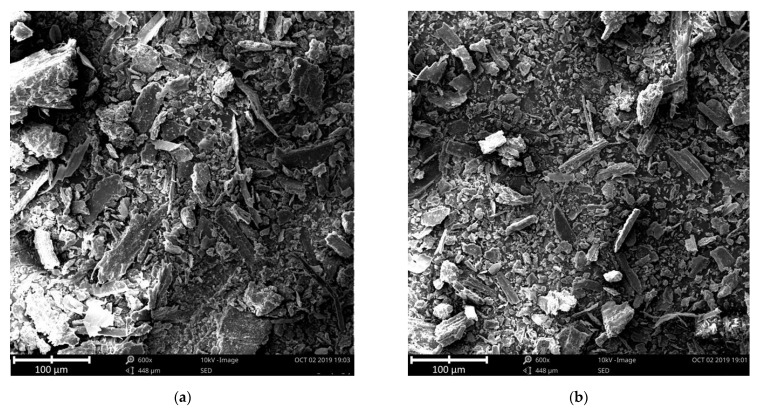
SEM images in 600× magnification of: (**a**) milled Type-1 straw dust; (**b**) milled Type-2 straw dust.

In the case of both straw types, straw dust was made under the same conditions mentioned above. The resulted dust was examined under SEM, and it showed that most of the particle size of dust was under 50 µm, but the overwhelming majority was under 5 µm, while the maximum diameter was 100 µm. The dust was sieved to remove all the particles that may have contained porosity itself; therefore, all the stem′s porosity had vanished (see Figure 3).

**Figure 4:**
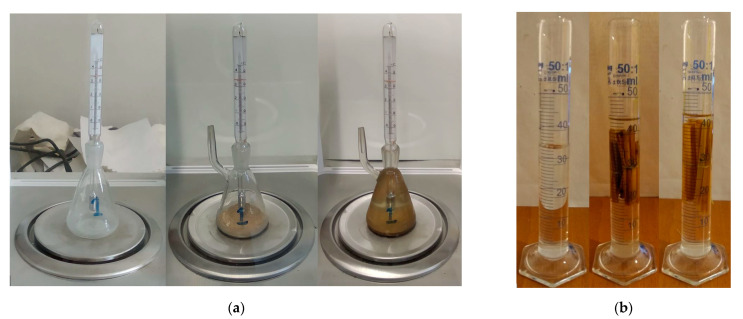
(**a**) Material density measurement with pycnometer, from left to right: empty pycnometer, straw dust added, straw dust and isopropyl alcohol added; (**b**) straw stem density measured with Archimedes method, from left to right: distilled water, Type-1 immersed, Type-2 immersed.

The material density of the straw using straw dust was measured using a Kimble pycnometer (DWK Life Sciences GmbH, Mainz, Germany) (Figure 4a), while the straw stem density was tested by the Archimedes method (Figure 4b). Only the tubular parts of the stem were measured; the nodes were excluded because they could make the results larger, and they would, therefore, not be representative for most of the fiber set. During the weighing of mass, the bundles of stems were completely dry. For the measurement of the volume of stems, they were completely water-saturated. From the material density and stem density, the porosity ($p_{f}$) of stems could be determined, which is an important input parameter in the modeling of the thermal conduction in fibers. The measuring tube is graded in milliliters; therefore, the measurement accuracy is ±0.5 mL, and it has an effect on stem density and on porosity, respectively. Thus, these two parameters are not a specific number but a relatively narrow interval.

**Figure 5:**
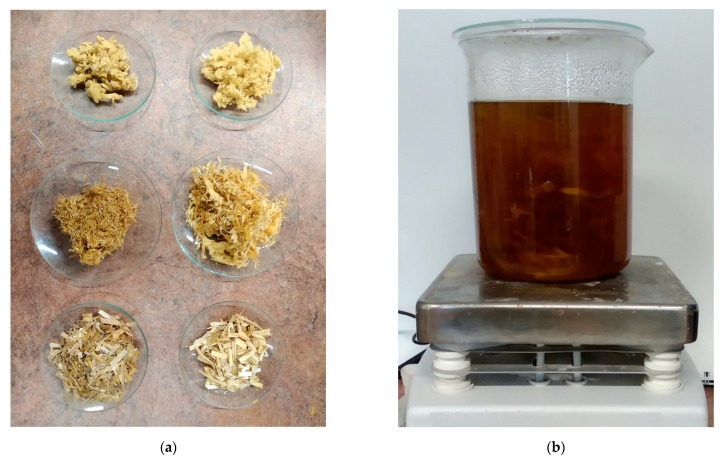
(**a**) Experimental determination of cellulose content, left column is Type-1, and the right column is Type-2 straw, from bottom to top: chopped raw straw, mixed fibers, residual fibers containing almost only clear cellulose; (**b**) separation of cellulose from other materials in NaOH solution.

An important input data for the model to calculate the thermal conductivity factor of the solid component is the cellulose content of the straws. To measure it, firstly, the stems were dried as in the case of determining their porosity (Figure 5a). Then, the cellulose component of stems was separated conventionally [55]. Chopped stems were put in thick NaOH (pH 14) solution and stirred at 100 °C (Figure 5b). The ratio of initial dry mass and residual fiber mass determines the cellulose content of the straw.

The diameter of the stems must be measured for the model, but it is decisive in many ways. The deviation of the stems′ measured diameter was large; therefore, 300 randomly selected pieces of stems were measured in two perpendicular directions for both types using a digital caliper with ±0.02 mm precision (Vogel Germany Gmbh & Co. KG, Kevelaer, Germany). In our proposed model, in the case of the diameter of the stems as an input parameter, only the mean value was used because we tried to keep the model as simple as possible. The most important definitions and all the symbols representing experimental values are listed in Appendix A.

#### 2.2.2. Thermal Conductivity Measurement

We measured the thermal conductivity of randomly oriented bulk raw straws according to the EN 12667 standard using the guarded hot plate method [56] with Taurus TLP 300 DTX equipment (Taurus Instruments GmbH, Weimar, Germany). Specimens were prepared according to EN 12667 and EN 1946-2 [57], respectively. The investigated bulk density range of the straw samples was between 80 and 180 kg/m^3^ in the case of both types, not only to calibrate or validate our model, but also to identify the optimal bulk density that gives the lowest possible thermal conductivity and can often be found for natural fibrous thermal insulations [20].

**Figure 6:**
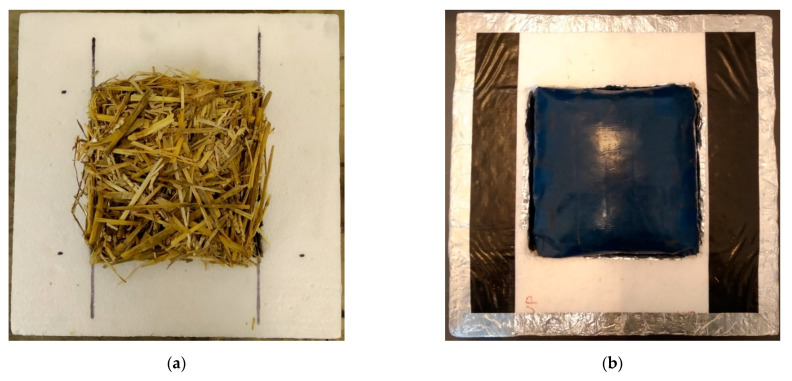
(**a**) Randomly oriented straw bulks; (**b**) closed measuring box filled with randomly oriented straw bulks.

At every bulk density, three samples were made and measured at three temperature steps (around 10 °C, 20 °C, and 30 °C mean temperatures) using a 10 K temperature difference. The results of the measurements were given at 10 °C sample mean temperature, since this temperature is given in ISO 10456 standard [58], respectively. In the experiments, the interval between the two densities was 20 kg/m^3^. The raw straw stems were filled to a closed thin-walled (<0.5 mm) PE foil box. The PE foil was penetrated to avoid inflation during compression. The foil makes a slight and constant difference in the test results. The stems were filled in a completely dry state randomly oriented into the box (Figure 6a). The size of the box was 150 mm × 150 mm × 50 mm. This box was taken into a stock made of EPS foam and aluminum sealing tape, ensuring that no moisture can penetrate the samples during the measurement (Figure 6b). Although the guarded hot plate method for testing thermal conductivity is more time-consuming than using a heat flow meter, it produces high precision measurements with excellent repeatability. Therefore, we obtained the experimental bulk density–thermal conductivity curves as precisely as possible to minimize the deviation by measurement, which is often present when measuring natural fibers [59]. Reducing the measurement deviation is essential because raw fibers naturally differ slightly, but these measurements served both for calibration (Type-1 straw) and for model validation (Type-2 straw); therefore, we wanted to keep the standard deviation of the measurements as low as possible and originated only from the stems, not from the measurement itself.

### 2.3. Analytic-Empirical Model Development

The reason that we must create a new model for straw-based fibrous thermal insulations is that we cannot apply any of the existing models to approximate the thermal conductivity of this composite structure. However, we used the previously mentioned three models as a base for our contribution. Two out of the three base models [45,46] deal with silicate-based solid fibers, and one of them deals with porous fibrous structures, although the structure is also significantly different from straw [26]. None of the previous models consider stem density or the effect of changing bulk density. Therefore, the existing models cannot describe the behavior of porous fiber sets. However, each cited model has a different connection with our studied material. One of them links to the connection system of the heat transfer models [46], while the other deals with the description of the heat transfer in the fibrous system in an understandable way [45,60]. The third model describes a system that is a fibrous and porous system at the same time as a straw stem [47], but in this case, the structure is reversed because the pores are inside the stems and not outside as in aerogel blankets.

**Figure 7:**
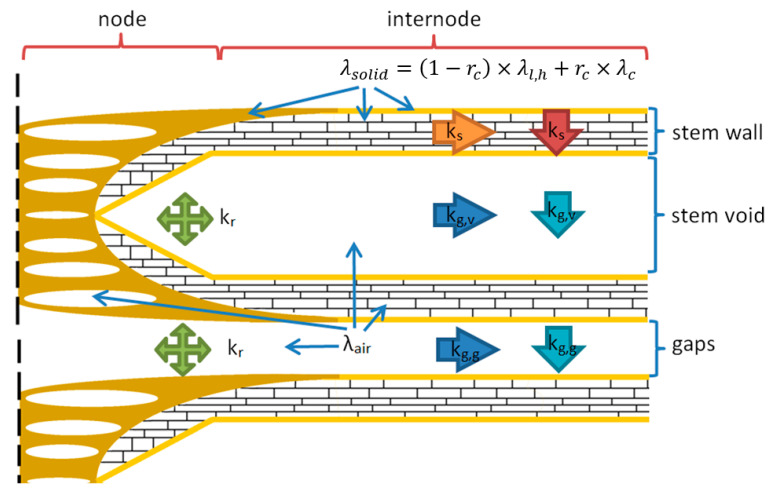
Components of heat transfer in the longitudinal section of straw-based fibrous media.

Straw stems are tubular cylinders, which contain voids and pores. In the specimens during the thermal conductivity measurements, almost all the stems were approximately perpendicular heat flow direction (see Figure 6). Therefore, we assumed that heat transfer occurs (if any point of the specimen is considered) in the direction of the stem from the warmer side until that stem contacts another fiber closer to its colder side. It then spreads to the next thread, and so on. We separated the four main components of heat transfer in a straw-based insulation material for our model (Figure 7):

1. k_s_—conduction through the solid medium (fibers), this component includes the heat transfer in the natural composite and in its embedded air pores;
2. k_g.tot_—conduction through gas medium including:
  - k_g.g_—conduction in the air trapped in gaps among the stems,
  - k_g.v_—conduction in the voids of stems;
3. k_r_—radiative heat transfer;
4. k_conv_—convection in the air in gaps between the fibers.

In our analytic-empirical model, we have neglected the effect of convection, since convection is generated by the movement of air molecules in the gaps or voids and due to the small dimensions of the gaps among stems. In addition, because walls roughly close the voids of stems (<3 mm), the convection effect is minor and practically negligible [61,62]. The ratio of radiation compared is an order of magnitude smaller compared to other conductive components in the investigated temperature range, similar to previous studies [60], although the radiation component has also been taken into consideration in our proposed model.

The assumptions used in our proposed analytic-empirical model are summarized as:

- Steady-state three-dimensional heat transfer occurs in the components of the material;
- Natural convection is negligible due to small pore and void sizes, and as a result, the air is static;
- In the case of conduction through the solid medium, we considered both natural composite components and entrapped air in the stems;
- Parallel heat transfer occurs between phases of the solid medium;
- With the growing bulk density, the dominance of gas and solid conduction should change in an inverse way;
- There is no heat generation source inside the medium (no negative heat transfer components);
- The model gives an accurate estimation only if the necessary and representative material properties of stems are measured in the laboratory;
- The model is applicable in the investigated bulk density range of 80–180 kg/m^3^;
- For creating the model, only the material properties and thermal conductivities of Type-1 straw were used. The material properties and measurement results of Type-2 were used only for validation of the model.

To simplify the presentation of the analytic-empirical model and its equations, we listed the most important definitions and all symbols used in Appendix A.

#### 2.3.1. Heat Transfer by Conduction in Stems

In the case of straw, heat-conduction in stems is a complex procedure because the stems are composite materials containing several sub-components. These are cellulose, hemicellulose, lignin, and air. Naturally, the composite also contains some other components, but the ratio of these phases is small enough to be neglected. This part of the model must take into consideration the conduction of the solid part of stems and conduction of gas-phase inside the pores of the stem wall. All the previous phases determine the thermal conductivity factor of the solid part. Based on Papadopoulos and Anastaselos′ model [45] from the bulk density of insulation, the stem density of fibers and material density are two important parameters that have to be defined; these are *f*_1_ and *f*_2_ in Equations (1) and (2):

(1) $$f_{1}=\frac{\rho_{ins}}{\rho_{f}}$$

(2) $$f_{2}=\frac{\rho_{f}}{\rho_{m}}$$

To be aware of every component of the solid material, the porosity ($p_{f}$) and the cellulose content ($r_{c}$) of stems must be known. The thermal conductivity of the actual (non-porous) solid part is calculated by the combination (based on the mass ratio of material phases) of the thermal conductivities of the components of the composite ($\lambda_{c},\;\lambda_{l,h}$). The conduction in the actual solid part of the stem can be calculated based on Equation (3). The air heat conduction setpoint was calculated as follows in Equation (4), where ξ≅2 for air:

(3) $$\lambda_{solid}=\left(1-r_{c}\right)\times \lambda_{l,h}+r_{c}\times \lambda_{c}$$

(4) $$L_{c}=\frac{\pi }{4}\times \frac{d_{a}}{\left(f_{1}+f_{2}\right)},\;s_{f}=69\;\mathrm{nm}\times \frac{P_{0}}{P}\times \frac{T}{T_{0}}\to Kn=\frac{s_{f}}{L_{c}}\to \lambda_{g}\;=\frac{0.021+8\times 10^{-5}\times T/K}{1+2\times \xi \times Kn}$$

After that, the solid conduction ($\lambda_{solid}$) and the entrapped gas conduction ($\lambda_{air}$) can be combined, based on the described equations about serial systems ($\lambda_{s,g}$) based on [46], see Equation (5):

(5) $$\lambda_{s,g}=\frac{\lambda_{solid}\times \lambda_{air}}{\left(1-p\right)\times \lambda_{air}+p\times \lambda_{solid}}$$

The overwhelming majority of the stems in the investigated straw bulks were parallel with the heated and the cooled surface during the thermal conductivity experiments, as presented earlier. Since the heat must travel perpendicular primarily to the measured surfaces, it passes primarily through the cross-section of the stem walls in a hypothetical path. It is assumed that the heat transfers in the shortest possible way (to reach the next stem). Voids serve as scattering centers for phonons (atomic vibrations); therefore, while heat flow transfers through solids and voids, respectively, gas conduction is much lower. The thermal conductivity factor through fibers changes with the bulk density of the insulation material.

**Figure 8:**
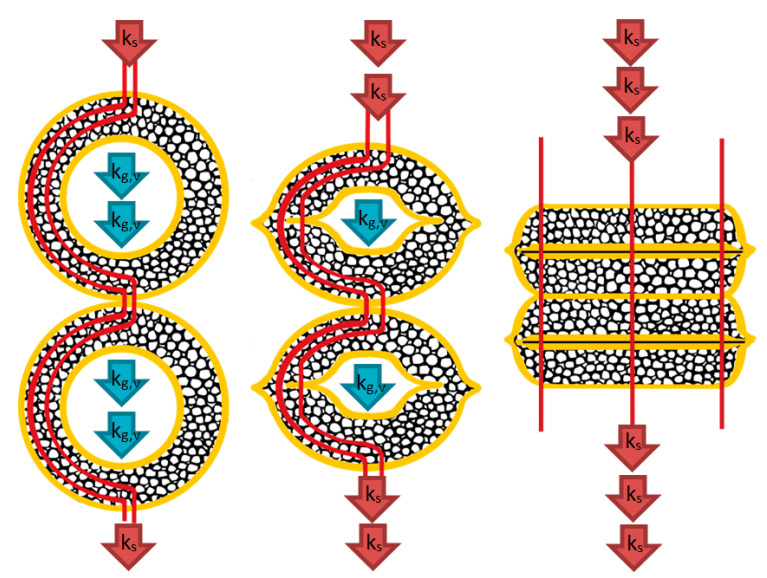
Effect of stem density on the length of heat path describing the idea of the flatness factor.

The stems are flattened during the compaction and become denser. Therefore, we can draw two consequences: firstly, the ratio of embedded air is decreasing (thus the ratio of solids is increasing), and secondly, while the stems are flattening, the heat can spread through a shorter path in the cross-section of the stem wall, so the conduction of solid phases becomes more significant (Figure 8). This effect can be handled by the introduction of a parameter that can handle it in our model, the flatness factor. Parallel with this phenomenon, since the the voids serve as scattering centers for phonons and the voids of stems also become smaller, the gas conduction factors become smaller as well ($k_{g.v}$), which must also be handled.

When the stem bulk is in an absolute loose state (uncompressed, natural density), the cross-section of stems is well approached by a circle. In this case, the shortest way for the heat is equal to the half perimeter of the stem in the centerline of the stem wall cross-section, which is described in Equation (6):

(6) $$s_{max}=(d_{a}-\frac{t_{w}}{2}\times \pi /2).$$

It is the longest heat path (absolute maximum), which means heat transfer needs more time because the heat must spread a long way. This state gives the minimum value of the thermal conductivity factor through fibers. Natural density is an experimental value that gives the bulk density without any compression (loose stem bulk which fits in the test/measurement box).

In a fully compressed state, the opposite points of the inner surface of the tubular stem are almost in contact in Equation (7):

(7) $$s_{min}=d_{min}\;\;$$

In this case, the shortest way for the heat is equal to the thickness of fully compressed stems; this value is also experimental, based on a large number of measurements. It is the shortest heat path (absolute minimum), which means heat transfer needs less time because the energy must spread in a short way. This state gives the maximum value of the thermal conductivity factor through fibers. With the use of linear interpolation between these values, the actual effect of heat′s path can be calculated.

The introduced flatness and flatness factor can handle this relationship between the length of the heat path (which is a function of stem density) and heat conduction through fibers in the model (Figure 8). In light of the above explanations, to obtain the flatness factor, we need to calculate ls, Vs, and ρins using Equation (8) to be able to obtain the flatness vector Equation (9), that assigns the shortest path to the lowest density and the longest path to the highest stem density, which will contribute to describing the resistance against heat flow in the stems:

(8) $$l_{s}=\frac{\rho_{ins}}{m_{l}}\to V_{s}=l_{s}\times \frac{d_{a}^{2}\times \pi }{4}\to p_{ins}=\frac{1\times m^{3}-V_{s}}{m^{3}},$$

(9) $$S_{fl}=s_{min}+\left(\rho_{ins}-\rho_{nat}\right)\times s_{max}-s_{min}/\left(\rho_{max}-\rho_{nat}\right).$$

Then the flatness factor, which is a vector of ratios, can be calculated with respect to the max length of the heat path using Equation (10):

(10) $$\mathrm{Fl}=\frac{S}{S_{max}}.$$

The thermal conductivity factor of the stem can be calculated from λ_s,g_, *f*_1_, *f*_2_, and D based on [45] using Equation (11):

(11) $$k_{s}=[{\left(f_{1}+f_{2}\right)}^{2}\times \lambda_{s,g}]\times D.$$

#### 2.3.2. Heat Transfer by Gas Conduction

Assuming that the air inside the straw stems is stationary, the thermal conductivity factor of the entrapped air by heat conduction of the stationary air ($\lambda_{g}$) is included in $\lambda_{s,g}$, respectively, to enable the model to consider the quantity of pores to solid in the stems depending on the compression of the stems.

The gas conduction in the bulk straw insulation material must be separated into two parts. The first part is the gas conduction factor in voids of stem ($k_{g.v}$) and the second part is the gas conduction factor in gaps ($k_{g.g}$) among stems. Both are based on the thermal conductivity of stationary air. In the case of the gas conduction factor in voids, the following assumption can be made: it has smaller and smaller values while the voids become tighter due to compression because the number of gas molecules in voids is decreasing, which thus collide less so transfer less heat to each other.

The compression of stems (increasing stem density) has a proportional but opposite effect to gas conduction in voids than the conductivity of the solid part in the stem wall. Therefore, the conduction factor will be proportional to the reverse of the flatness factor (which can be formed using an exchange matrix) as shown in Equation (12):

(12) $${\mathrm{Fl}}_{rev}=\mathrm{Fl}\times \left(\begin{array}{ccc} 0 & \cdots & 1\\ \vdots & \ddots & \vdots \\ 1 & \cdots & 0 \end{array}\right).$$

Although the flatness factor basically is related to the deformation of the stem wall and the thermal conductivity of the solid part, some modification of this factor must be applied to describe the gas thermal conduction changes in voids because the voids inside the stem is an inseparable part of the stem. Therefore, it is foreseeable while the thermal conductivity factor through fibers increases as a result of the compression, then the gas conduction factor in voids of the stem is decreases (see Figure 8) which can be described using Equation (13)):

(13) $$k_{g.v}=\lambda_{g}\times {\mathrm{Fl}}_{rev}.$$

The second part is the gas conduction factor in gaps among stems. The value of this factor is based on a similar principle as the previous one. This value is much less measurable due to material particularities; therefore, it was determined based on theoretical calculations. If the ratio of gaps is decreasing, then the bulk density of insulation material is increasing. If the bulk density of the raw straw insulation material, the unit mass of stem by length (g/mm), and the average diameter are known, the volume ratio of the stems is also determinable. From these values, the volume ratio of gaps (*ρ_ins_*) can be calculated, which we also used to obtain the flatness factor, and the resulting vector will be proportional to the gas conduction factor among gaps, as Equation (14) describes:

(14) $$k_{g.g}=\lambda_{g}\times p_{ins}.$$

#### 2.3.3. Total Heat Transfer by Conduction

Both conduction factors approach zero while the stem density of the insulation is increasing because the gas medium is displaced from the material (see Figure 8), and the conduction of the solid part become dominant. The sum of the solid and gas conduction factors gives the total heat transfer by conduction ($k_{c,tot}$) in the investigated material and can be calculated using Equation (15):

(15) $$k_{c,tot}=k_{s}+k_{g.v}+k_{g.g}.$$

#### 2.3.4. Heat Transfer by Radiation

There are analytical methods available to calculate the radiative heat flux. In most cases, a complex system of simultaneous differential and integral equations must be used. One of the standard methods to calculate the radiative thermal conductivity is the Rosseland equation. When this equation is applied, the spectral mean extinction coefficient and must be known or measured. Fukushima and Hatfield [63] measured the extinction coefficient of different cereals, separated the leaf and the stem. In our research, the specimens contained both stems and leaves, so the applied extinction coefficient is the mean of leaf and stem, *β* = 1865 m^2^/kg. Based on the specific extinction coefficient and the extinction coefficient (*β*), the heat transfer by radiation ($k_{r}$) can be calculated using Equations (16) and (17) based on [45]:

(16) $$\beta =\frac{\left(\beta_{leaf}+\beta_{stem}\right)}{2},$$

(17) $$k_{r}=\frac{16\times \sigma \times T^{3}}{3\times \beta }\times {\mathrm{Fl}}^{3}.$$

#### 2.3.5. Total Thermal Conductivity

Finally, the total value of the heat transfer is summarized in the total thermal conductivity factor ($k_{tot}$) that can be defined by the sum of the conduction and radiation factors using Equation (18):

(18) $$k_{tot}=k_{c,tot}+k_{r}.$$

## 3. Results and Discussion

### 3.1. Physical Properties of Straw Stems

The experimentally measured material properties are summarized in Table 1. Type-1 material physical properties were used to create our proposed model, while Type-2 properties were used only for validation purposes afterwards. As is visible in Table 1, stem density is greater for Type-2 than Type-1 straw by more than 11.5%, while material density is the other way around: Type-1 is larger by 12% than Type-2. This shows a reversed trend that can be explained by the porosity of the two different straw materials. Type-1 straw is more porous, as can be seen in the SEM images in Figure 1. The difference in porosity is 3.9% in favor of Type-1.

The other significant difference in the material properties comparing Type-1 and Type-2 straw is their cellulose content. Our wheat samples had a very similar percentage of cellulose reported in [54], while barley had almost 5% lower cellulose content. Since these two straws are from different vegetables, the difference is understandable. This difference indicates that these two straws will have different thermal conductivity.

The average diameter of the examined 300 pieces of randomly sampled straw stems showed that Type-1 has a slightly larger average stem diameter than Type-2. However, as we discussed earlier, as explained in Figure 1, Type-2 straws have thicker stem walls.

### 3.2. Thermal Conductivity of Straw Bulks

**Figure 9:**
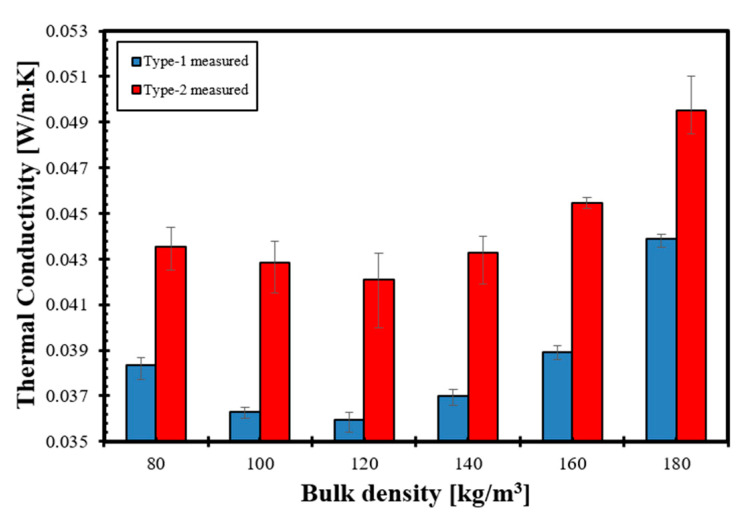
Measured thermal conductivity of randomly oriented Type-1 and Type-2 straw bulks with different bulk densities.

The thermal conductivity measurement results are summarized in Figure 9, which shows the average thermal conductivity and the upper and lower values. The average standard deviation of the measurements was 0.0004 W/m·K for Type-1 and 0.0011 W/m·K for Type-2. Both straw types’ standard deviations can be considered acceptable, and the slightly higher standard deviation for Type-2 can be explained with its pore system, which showed greater variance than Type-1, as shown earlier in Figure 1.

As expected, Type-1 straw has smaller thermal conductivity than Type-2 due to its higher porosity and lower cellulose content. Both straw types show a similar trend in terms of changing bulk density, and we measured the lowest thermal conductivity for both Type-1 and Type-2 straw bulks at 120 kg/m^3^ bulk density, which can be considered the optimum bulk density. This optimum bulk density is higher than that of mineral wool or bagasse (100 kg/m^3^), hemp or palm fiber (90 kg/m^3^), flax fiber (80 kg/m^3^), feather fiber (60 kg/m^3^) or wood wool (50 kg/m^3^), respectively [20,50,64,65].

Compared to other studies dealing with raw straw materials, our investigated straw types′ measured bulk density-dependent thermal conductivities are lower than what Conti et al. [30,66] or Costes et al. [31] measured on full-size straw bale walls that were filled with compressed straw bales oriented in parallel and made of different straws, which were probably also more humid than our laboratory conditioned samples, but in the same range that the literature review of Costes et al. [31] showed for straw bales with fibers oriented perpendicular to the heat flow.

### 3.3. Validation of the Analytic-Empirical Model

**Figure 10:**
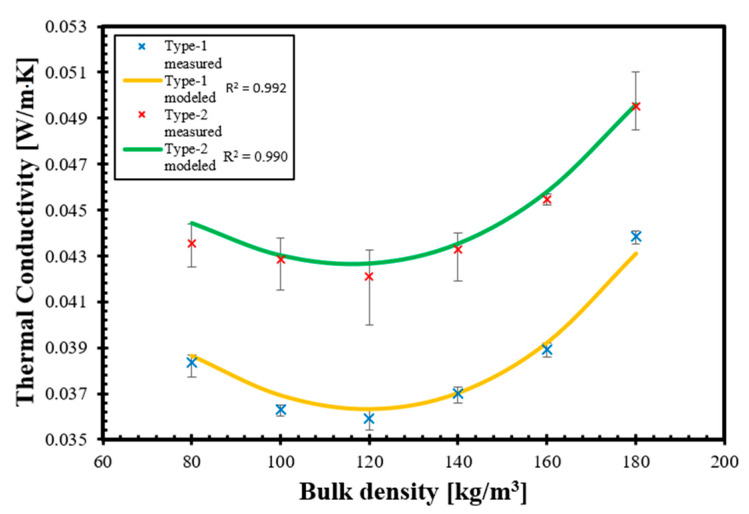
Relationship between bulk density and total thermal conductivity in case of the results of laboratory measurements and the analytic-empirical model using Type-1 and Type-2 straws.

Figure 10 shows the measured thermal conductivity and the results of the analytic-empirical model. As we mentioned, we created the model using only the physical properties of Type-1 straw. When the model produced a value of R^2^ ≥ 0.99, we tested the calculation using the measured material properties of Type-2 straw. The validation showed that our model, fed with the properties of Type-2 straw, obtained a 0.99 R^2^ value. Therefore, we considered it acceptable. As is visible in Figure 10, the largest difference between the measured and modeled values is less than 2%. This difference could be due to the impact of injury level of the stems, which needs to be investigated in the future, and, including these integrity effects, we are probably able to increase the accuracy of our model. It is also observable in Figure 10 that our model′s characteristics and trends match with the measured results, and the curve of bulk density versus thermal conductivity calculated using our model reasonably matches the experimentally measured results within the investigated bulk density range.

### 3.4. Bulk Density Dependency of the Thermal Conductivity Factors

**Figure 11:**
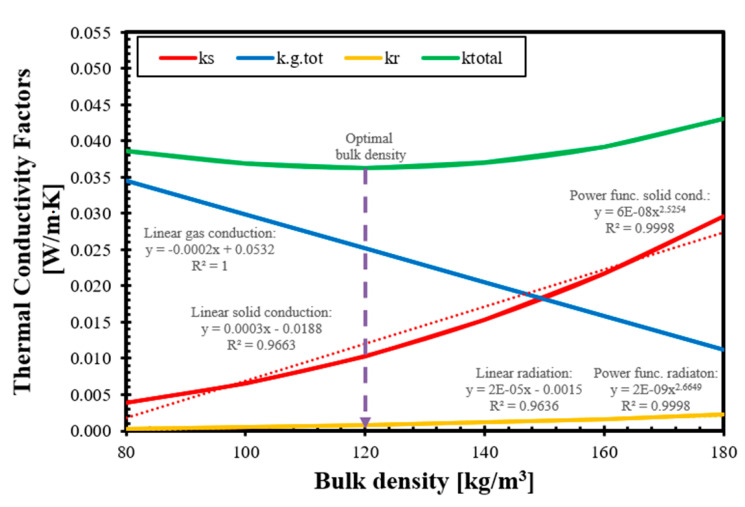
Thermal conductivity factors from the model in relation to the function of bulk density with added trend lines, regression equations, and R^2^; Type-1 straw.

**Figure 12:**
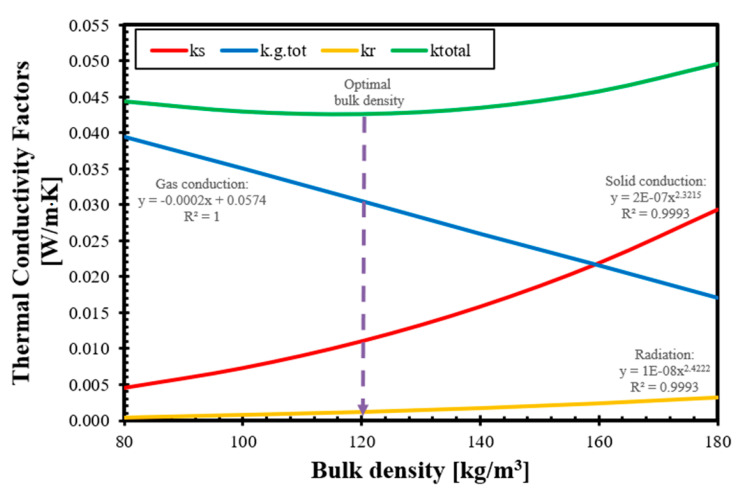
Thermal conductivity factors from the model in function of bulk density; Type-2 straw.

The total thermal conductivity factor of Type-1 and Type-2 straw using our validated analytic-empirical model can be calculated using Equation (18), and the results and its components are shown in Figure 11 for Type-1 straw and Figure 12 for Type-2 straw. In the figures, we also included trend lines that fit with R^2^ ≥ 0.99 to the model results of each component.

These trend lines show that the thermal conductivity factor of gas conduction decreases linearly in the function of bulk density, and it is consistent with the expectations, while the solid conduction and radiation thermal conductivity factors change exponentially with the increase in bulk density. In the investigated bulk density range, the gas conduction factor could be approximated using a linear function with a perfect R^2^ value, while the radiation factor could be reached using a linear function with R^2^ values of 0.964 and 0.968 for Type-1 and Type-2 straws, respectively, and these approximations would not have significantly impaired the accuracy of the final results since radiation is accountable only for 0.67% to 6.43% of the total thermal conductivity, depending on the bulk density at 10 °C and dry state, as it was proven earlier that the radiation component is negligible at low temperatures for fibrous thermal insulations [60], and the bulk density dependence shows only a slightly curved trend line [62].

In the present model, the value of the radiative component ($k_{r}$) is regulated by the cubic function of the flatness factor, but it also has a very small curvature at 10 °C. However, if we approximate the solid conduction using linear trend lines, despite the fact that we can achieve a 0.966 and 0.971 R^2^ fit, the results were visibly different in the case of the gas conduction factor, and the increase could reach up to 71% at 100 kg/m^3^ bulk density and, in the investigated range, an average of 46.5% for Type-1 and 37.9% for Type-2. Therefore, we state that the trend of the solid conductivity factor can only be modeled using a power function, and only this type of regression can give high accuracy. We included all the above-mentioned trend lines and their equations in Figure 11, and showed Type-1 straw while showing only the acceptable trend lines in for Type-2 Figure 12.

It is also observable in the figures that the value of the solid thermal conductivity factor is growing as the bulk density is increasing because the ratio of solids in the natural composite (which has higher thermal conductivities than still air) is growing. We must note that the value of the gas conduction factor changes in the opposite way and with a simpler trend than the solid conduction factor. It is decreasing while the bulk density of insulation is growing. This is because, in the thermal conductivity factor of gas conduction, we included the gas components depending on the bulk density of the straw thermal insulation, and not only the thermal conductivity of stationary air, although that is also a roughly constant value at 10 °C. Therefore, the behavior of the linear curve is understandable because the quantity of the air (in gaps and voids) is decreasing where heat conduction can occur if we increase the stem and bulk density, and therefore, the amount of solid component in bulk. Theoretically, gas conduction could be reduced to zero when all the gases are squeezed out from the insulation.

The obtained trends of the thermal conductivity factors are different from what we used to obtain with conventional heat transfer models and conventional thermal insulation materials since, in materials such as mineral wool or organic foams, the total heat transfer is dominated by the contribution of the gas conduction within the hollow spaces or pores [67], while in our model, the gas conduction decreases rapidly with the increase in the bulk density. We can also compare the trends and similarities of the thermal conductivity factors obtained by our model for straw-based materials, depending on their density, with a different heat transfer model that has recently been published [68]. This other new model describes the heat transfer in recycled glass foams and also shows thermal conductivity factors separately. This model is different from ours in terms of the description of solid and gas conduction (since glass foam is significantly different from natural fibrous materials), but it used the Rosseland equation to include radiation, similarly to our model. Comparing their results, the solid and gas conduction shows very similar trends. Still, the radiation factor is decreasing with an increasing density, similarly to most conventional insulations or vacuum panels [62].

If we compare our model results for Type-1 and Type-2 straws, it is clear that while the trends and the characteristics of the curves are similar, the main difference between barley and wheat straw is the gas conduction, which is due to the different pore structure of the stems, and there are only slight differences in the solid conduction and radiation factors. We can state that the most important physical property to create a good thermal insulator material out of a straw is the pore structure of the crop.

## 4. Conclusions

In the present paper, a validated analytic-empirical model was presented, which was developed to describe the heat transfer process in raw straw bulks, depending on their bulk density, in the range of 80–180 kg/m^3^, and the physical properties of different straw stems with the assumptions described in Section 2.3. The following conclusions can be drawn from our research:

- From the relationship between bulk density and thermal conductivity, it is observable that the characteristic of the curve is independent of the material properties of the different investigated straws (barley and wheat). The absolute value of the thermal conductivity is regulated by the inner structure and the physical properties of individual stems. Therefore, with different inner compositions, a shifted version of similarly shaped curve characteristics can be obtained. The most important parameter is the porosity of the stems. Our model is sensitive to the physical properties of the straw stems and only applicable for the calculation of the thermal conductivity of straw fiber thermal insulation bulks if the material properties of the used straw are measured and defined.
- In our paper, we demonstrated two types of straws and their physical properties, barley and wheat straw, as well as their thermal conductivity depending on the bulk density. The optimal bulk densities of both straw types were found to be similar, at 120 kg/m^3^.
- The thermal conductivity factor of straw bulks must be separated into three main components: conduction in solids, conduction in gases, and radiation. These are the same components as in the case of every fibrous material. However, the gas conduction in straw insulations should be separated into another three subcomponents, which are: conduction in the pore gas of the stem, conduction in the voids of the stem, and conduction in the gaps among the stems.
- The individual stem is a natural composite material that contains different solid material components. The thermal conductivity of the stem′s solid part can be calculated from the product of the components′ thermal conductivities and the mass ratio of the components. For simplification, differentiating only cellulose and other solid components did not cause a significant difference in the model calculations; therefore, obtaining only the cellulose content of a straw type provides sufficient data for model calculations.
- The individual stem not only contains different solid material components but also different phases. In the light of heat transfer in individual stems, the gas and solid phases can be modeled as they are connected in parallel. The value of the individual systems can be modeled by knowing the components′ thermal conductivity and the volume ratio of the components, for which we can use porosity. We also proved that the porosity of the stems is the most important material property to be known in the case of straw-based fibrous thermal insulations.
- The path of the heat flow in the stems′ cross-section is changed by the stem density of the straw fibers. A vector derived from the length of this path, called the flatness factor, is introduced to our model, and this can control the value of heat conduction in the stem wall and in voids with accurate precision.
- The trends of thermal conductivity factors on bulk density, depending on thermal conductivity, are observable, and while the gas conduction shows the linear trend and decreases with density, solid conduction and radiation increases exponentially with bulk density and can be described using power functions with high accuracy. However, radiation may be simplified to a linear model in the investigated bulk density and temperature range without a loss of precision.

## Figures and Tables

**Table 1:** Measured physical properties of the investigated straw types.

Straw Stem	Stem Density (g/cm^3^)	Material Density (g/cm^3^)	Porosity (1)	Cellulose Content (%)	Average Stem Diameter (mm)
Type-1	0.190	1.3197	0.852	37.5	3.15
Type-2	0.212	1.1770	0.820	42.4	3.02

## Data Availability

The data presented in this study are available on request from the corresponding author.

## Appendix A

### Appendix A.1. Definitions

Flatness factor: this is a vector that shows the dominance of the thermal conductivity of the solid part of a stem at the current density compared to the natural and maximum density.

Gaps: spaces among individual stems in the compressed bulk of the stems.

Material density: density of the stems without pores and voids.

Maximum density: the density values at which all of the fibers are fully compressed. The inner surface of the stem wall is almost in touch.

Natural density: experimental value, gives bulk density of the insulation without any compression (loose stem bulk which is fitted into the test box).

Pore: volume inside the stem wall which contains gases or liquid.

Reverse flatness factor: this is a vector that shows the dominance of thermal conductivity of the gas in the voids inside stems at the current density compared to the natural and maximum density.

Stem density: density of stems that contain pores, without voids.

Void: volume in the tubular stem, which is bounded by the inner side of the stem wall and nodes.

### Appendix A.2. Nomenclature of Material Properties

d_a_: average diameter of stems.

p: porosity.

r_c_: cellulose content.

t_w_: the thickness of the stem wall.

ρ_f_: stem density.

ρ_ins_: bulk density of the straw specimen.

ρ_m_: material density.

### Appendix A.3. Nomenclature of Experimental Values

d_min_: the thickness of the fully compressed fiber.

m_l_: unit length stem mass.

ρ_max_: fully compressed specimen′s bulk density.

ρ_nat_: uncompressed specimen′s natural density.

### Appendix A.4. Nomenclature of Literature Data

d_g_: collision diameter of the air.

K_B_: Boltzmann′s constant.

s_f_: mean free path of air molecules.

β_leaf_: extinction coefficient of steam.

β_leaf_: extinction coefficient of steam′s leaf.

λ_c_: thermal conductivity of cellulose.

λ_h_: thermal conductivity of hemicellulose.

λ_l_: thermal conductivity of lignin.

σ: Stefan–Boltzmann constant.

### Appendix A.5. Nomenclature of Model Parameters

f_1_: density ratio of ρ_ins_ and ρ_f_.

f_2_: density ratio of ρ_f_ and ρ_m_.

F_l_: flatness factor.

F_lrev_: reverse flatness factor.

k_c,tot_: total conductivity factor.

k_conv_: convection factor.

k_g.g_: gas conductivity factor in the gaps among stems.

k_g.tot_: total gas conductivity factor.

k_g.v_: gas conductivity factor in the voids of stems.

k_r_: radiation factor.

k_s_: thermal conductivity factor of the stem.

k_tot_: total thermal conductivity.

L_c_: characteristic length.

l_s_: sum length of stems in the specimen.

p_ins_: volume ratio of gaps in the specimen.

S_fl_: flatness.

s_max_: maximum length of heat flow.

s_min_: minimum length of heat path.

V_s_: sum gross volume of stems in the specimen.

λ_g_: thermal conductivity of still air.

λ_s,g_: thermal conductivity of the stem.

f_2_: density ratio of ρ_f_ and ρ_m_.

### Appendix A.6. Nomenclature of Environmental Conditions

P: pressure.

RH: relative humidity.

T: temperature.

## References

[1] Varriale F. (2016). Forecasting future demand for domestic thermal insulation in Wales. Indoor Built Environ..

[2] Kotzeva M., Brandmüller T., Önefors A. (2020). Eurostat Regional Yearbook 2020.

[3] Ostańska A. (2018). Thermal Imaging for Detection of Defects in Envelopes of Buildings in Use: Qualitative and Quantitative Analysis of Building Energy Performance. Period. Polytech. Civ. Eng..

[4] Kosiński P., Wojcik R., Semen B. (2019). Experimental study on the deterioration of thermal insulation performance due to wind washing of the cavity insulation in leaky walls. Sci. Technol. Built Environ..

[5] Nagy B. (2019). Designing insulation filled masonry blocks against hygrothermal deterioration. Eng. Fail. Anal..

[6] Choi H.-J., Kang J.-S., Huh J.-H. (2018). A Study on Variation of Thermal Characteristics of Insulation Materials for Buildings According to Actual Long-Term Annual Aging Variation. Int. J. Thermophys..

[7] Gaujena B., Agapovs V., Borodinecs A., Strelets K. (2020). Analysis of Thermal Parameters of Hemp Fiber Insulation. Energies.

[8] Bull J. Embodied Carbon of Insulation. https://www.greenspec.co.uk/building-design/embodied-carbon-of-insulation.

[9] Zieger V., Lecompte T., de Menibus A.H. (2020). Impact of GHGs temporal dynamics on the GWP assessment of building materials: A case study on bio-based and non-bio-based walls. Build. Environ..

[10] Dong K., Jiang H., Sun R., Dong X. (2019). Driving forces and mitigation potential of global CO_2_ emissions from 1980 through 2030: Evidence from countries with different income levels. Sci. Total Environ..

[11] Johnston L., Hausman E., Biewald B., Wilson R. (2011). 2011 Carbon Dioxide Price Forecast.

[12] Shue H. (2017). Responsible for what? Carbon producer CO2 contributions and the energy transition. Clim. Chang..

[13] Fan M., Fu F. (2016). Advanced High Strength Natural Fibre Composites in Construction.

[14] Onuaguluchi O., Banthia N. (2016). Plant-based natural fibre reinforced cement composites: A review. Cem. Concr. Compos..

[15] Bouasker M., Belayachi N., Hoxha D., Al-Mukhtar M. (2014). Physical Characterization of Natural Straw Fibers as Aggregates for Construction Materials Applications. Materials.

[16] He J., Kawasaki S., Achal V. (2020). The Utilization of Agricultural Waste as Agro-Cement in Concrete: A Review. Sustainability.

[17] Bozsaky D. (2019). Nature-Based Thermal Insulation Materials From Renewable Resources—A State-Of-The-Art Review. Slovak J. Civ. Eng..

[18] Rojas C., Cea M., Iriarte A., Valdés G., Navia R., Cárdenas-R J.P. (2019). Thermal insulation materials based on agricultural residual wheat straw and corn husk biomass, for application in sustainable buildings. Sustain. Mater. Technol..

[19] Kristak L., Ruziak I., Tudor E., Barbu M., Kain G., Reh R. (2021). Thermophysical Properties of Larch Bark Composite Panels. Polymers.

[20] Dieckmann E., Onsiong R., Nagy B., Sheldrick L., Cheeseman C. (2021). Valorization of Waste Feathers in the Production of New Thermal Insulation Materials. Waste Biomass Valorization.

[21] Souza A.M., Nascimento M.F., Almeida D.H., Silva D., Almeida T.H., Christoforo A.L., Lahr F.A. (2018). Wood-based composite made of wood waste and epoxy based ink-waste as adhesive: A cleaner production alternative. J. Clean. Prod..

[22] Cellulose Fiber Market Size & Share, Industry Report, 2018–2025. https://www.grandviewresearch.com/industry-analysis/cellulose-fibers-market.

[23] Korjenic A., Petránek V., Zach J., Hroudová J. (2011). Development and performance evaluation of natural thermal-insulation materials composed of renewable resources. Energy Build..

[24] Koh C.H., Kraniotis D. (2020). A review of material properties and performance of straw bale as building material. Constr. Build. Mater..

[25] Abu-Jdayil B., Mourad A.-H., Hittini W., Hassan M., Hameedi S. (2019). Traditional, state-of-the-art and renewable thermal building insulation materials: An overview. Constr. Build. Mater..

[26] Vanova R., Vlcko M., Stefko J. (2021). Life Cycle Impact Assessment of Load-Bearing Straw Bale Residential Building. Materials.

[27] Sabapathy K., Gedupudi S. (2019). Straw bale based constructions: Measurement of effective thermal transport properties. Constr. Build. Mater..

[28] Platt S., Maskell D., Walker P., Laborel-Préneron A. (2020). Manufacture and characterisation of prototype straw bale insulation products. Constr. Build. Mater..

[29] Yang L., Yang J., Liu Y., An Y., Chen J. (2021). Hot box method to investigate U-values for straw bale walls with various structures. Energy Build..

[30] Conti L., Barbari M., Monti M. (2016). Steady-State Thermal Properties of Rectangular Straw-Bales (RSB) for Building. Buildings.

[31] Costes J.-P., Evrard A., Biot B., Keutgen G., Daras A., Dubois S., Lebeau F., Courard L. (2017). Thermal Conductivity of Straw Bales: Full Size Measurements Considering the Direction of the Heat Flow. Buildings.

[32] Cornaro C., Zanella V., Robazza P., Belloni E., Buratti C. (2020). An innovative straw bale wall package for sustainable buildings: Experimental characterization, energy and environmental performance assessment. Energy Build..

[33] Liu L., Zou S., Li H., Deng L., Bai C., Zhang X., Wang S., Li N. (2019). Experimental physical properties of an eco-friendly bio-insulation material based on wheat straw for buildings. Energy Build..

[34] Wang H., Chiang P.-C., Cai Y., Li C., Wang X., Chen T.-L., Wei S., Huang Q. (2018). Application of Wall and Insulation Materials on Green Building: A Review. Sustainability.

[35] Tobon A.M., Andres Y., Locoge N. (2020). Impacts of test methods on the assessment of insulation materials’ resistance against moulds. Build. Environ..

[36] Thomson A., Walker P. (2014). Durability characteristics of straw bales in building envelopes. Constr. Build. Mater..

[37] Yin X., Dong Q., Lawrence M., Maskell D., Yu J., Sun C. (2020). Research on Prediction Model for Durability of Straw Bale Walls in Warm (Humid) Continental Climate—A Case Study in Northeast China. Materials.

[38] Csanády D., Fenyesi O. Development of new-generation, eco-friendly thermal insulation board. Proceedings of the 13th FIB International PhD Symposium in Civil Engineering.

[39] Faruk O., Bledzki A.K., Fink H.-P., Sain M. (2012). Biocomposites reinforced with natural fibers: 2000–2010. Prog. Polym. Sci..

[40] Virk A.S., Hall W., Summerscales J. (2010). Failure strain as the key design criterion for fracture of natural fibre composites. Compos. Sci. Technol..

[41] Torres J.P., Vandi L.-J., Veidt M., Heitzmann M.T. (2017). The mechanical properties of natural fibre composite laminates: A statistical study. Compos. Part A Appl. Sci. Manuf..

[42] Shi L., Zhang H., Li Z., Man X., Wu Y., Zheng C., Liu J. (2018). Analysis of moisture buffering effect of straw-based board in civil defence shelters by field measurements and numerical simulations. Build. Environ..

[43] Gallegos-Ortega R., Magaña-Guzmán T., Reyes-López J.A., Romero-Hernández M.S. (2017). Thermal behavior of a straw bale building from data obtained in situ. A case in Northwestern México. Build. Environ..

[44] Balador Z., Gjerde M., Isaacs N., Imani M. (2019). Thermal and Acoustic Building Insulations from Agricultural Wastes. Handbook of Ecomaterials.

[45] Karamanos A., Papadopoulos A., Anastasellos D. Heat transfer phenomena in fibrous insulating materials. Proceedings of the 2004 WSEAS/IASME International Conference on Heat and Mass.

[46] Xie T., He Y.L., Li Y.S., Tao W.Q. Theoretical and Numerical Study on Thermal Properties of Fibrous Insulation Materials. Proceedings of the 14th Minsk International Forum on Heat and Mass Transfer.

[47] Hoseini A., McCague C., Andisheh-Tadbir M., Bahrami M. (2016). Aerogel blankets: From mathematical modeling to material characterization and experimental analysis. Int. J. Heat Mass Transf..

[48] Szagri D., Nagy B. (2021). Experimental and numerical hygrothermal analysis of a refurbished double-skin flat roof. Case Stud. Therm. Eng..

[49] Sonderegger W., Niemz P. (2012). Thermal and moisture flux in soft fibreboards. Eur. J. Wood Wood Prod..

[50] Kosiński P., Brzyski P., Suchorab Z., Łagód G. (2020). Heat Losses Caused by the Temporary Influence of Wind in Timber Frame Walls Insulated with Fibrous Materials. Materials.

[51] Gößwald J., Barbu M.-C., Petutschnigg A., Tudor E. (2021). Binderless Thermal Insulation Panels Made of Spruce Bark Fibres. Polymers.

[52] Loix C., Huybrechts M., Vangronsveld J., Gielen M., Keunen E., Cuypers A. (2017). Reciprocal Interactions between Cadmium-Induced Cell Wall Responses and Oxidative Stress in Plants. Front. Plant Sci..

[53] Alonso D.M., Wettstein S.G., Dumesic J.A. (2012). Bimetallic catalysts for upgrading of biomass to fuels and chemicals. Chem. Soc. Rev..

[54] Amezcua-Allieri M.A., Aburto J. (2018). Conversion of Lignin to Heat and Power, Chemicals or Fuels into the Transition Energy Strategy. Lignin—Trends and Applications.

[55] Watkins D., Nuruddin M., Hosur M., Tcherbi-Narteh A., Jeelani S. (2015). Extraction and characterization of lignin from different biomass resources. J. Mater. Res. Technol..

[56] Hungarian Standard Institute (2001). MSZ EN 12667:2001. Thermal Performance of Building Materials and Products: Determination of Thermal Resistance by Means of Guarded Hot Plate and Heat Flow Meter Methods: Products of High and Medium Thermal Resistance.

[57] Hungarian Standard Institute (1999). MSZ EN 1946-3:1999. Thermal Performance of Building Products and Components-Specific Criteria for the Assessment of Laboratories Measuring Heat Transfer Properties—Part 3: Measurements by Heat Flow Meter Method.

[58] Hungarian Standard Institute (2008). MSZ EN ISO 10456:2008. Building Materials and Products. Hygrothermal Properties. Tabulated Design Values and Procedures for Determining Declared and Design Thermal Values (ISO 10456:2007).

[59] Kosiński P., Brzyski P., Duliasz B. (2018). Moisture and wetting properties of thermal insulation materials based on hemp fiber, cellulose and mineral wool in a loose state. J. Nat. Fibers.

[60] Karamanos A., Hadiarakou S., Papadopoulos A. (2008). The impact of temperature and moisture on the thermal performance of stone wool. Energy Build..

[61] Wei G., Liu Y., Zhang X., Yu F., Du X. (2011). Thermal conductivities study on silica aerogel and its composite insulation materials. Int. J. Heat Mass Transf..

[62] Song K., Mukhopadhyaya P. (2016). Vacuum insulation panels (VIPS) in building envelope constructions: An overview. Int. Rev. Appl. Sci. Eng..

[63] Fukushima R.S., Hatfield R.D. (2004). Comparison of the Acetyl Bromide Spectrophotometric Method with Other Analytical Lignin Methods for Determining Lignin Concentration in Forage Samples. J. Agric. Food Chem..

[64] Grohe B. (2004). Heat conductivities of insulation mats based on water glass bonded non-textile hemp or flax fibres. Holz als Roh Werkstoff.

[65] Anh L.D.H., Pásztory Z. (2021). An overview of factors influencing thermal conductivity of building insulation materials. J. Build. Eng..

[66] Conti L., Goli G., Monti M., Pellegrini P., Rossi G., Barbari M. (2017). Simplified Method for the Characterization of Rectangular Straw Bales (RSB) Thermal Conductivity. IOP Conf. Ser. Mater. Sci. Eng..

[67] Simmler H., Brunner H., Heinemann U., Schwab H., Kumaran K., Mukhopadhyaya P., Quénard D., Kücükpinar-Niarchos E., Stramm C., Tenpierik M. (2005). Vacuum Insulation Panels—Study on VIP-Components and Panels for Service Life Prediction of VIP in Building Applications (Subtask A).

[68] Cimavilla-Román P., Villafañe-Calvo J., López-Gil A., König J., Rodríguez-Perez M. (2021). Ángel Modelling of the mechanisms of heat transfer in recycled glass foams. Constr. Build. Mater..

